# What lies on macroalgal surface: diversity of polysaccharide degraders in culturable epiphytic bacteria

**DOI:** 10.1186/s13568-022-01440-8

**Published:** 2022-07-27

**Authors:** Marta Barbato, Violetta Vacchini, Aschwin H. Engelen, Giovanni Patania, Francesca Mapelli, Sara Borin, Elena Crotti

**Affiliations:** 1 Dipartimento di Scienze per gli Alimenti, la Nutrizione e l’Ambiente (DeFENS), Università degli Studi di Milano, via Celoria 2, 20133 Milano, Italy; 2 Centro de Ciências do Mar (CCMAR), Universidade do Algarve, Campus de Gambelas, 8005-139 Faro, Portugal; 3 Present Address: Department of Biology, Section for Microbiology, Aarhus University, Ny Munkegade 116, 8000 Aarhus, Denmark

**Keywords:** Blue biotechnology, Epibiota, Seaweeds, Bioprospecting, Cultivable microbiota, Polysaccharidases

## Abstract

Macroalgal surface constitutes a peculiar ecological niche and an advantageous substratum for microorganisms able to degrade the wide diversity of algal glycans. The degrading enzymatic activities of macroalgal epiphytes are of paramount interest for the industrial by-product sector and biomass resource applications. We characterized the polysaccharide hydrolytic profile of bacterial isolates obtained from three macroalgal species: the red macroalgae *Asparagopsis taxiformis* and *Sphaerococcus coronopifolius* (*Rhodophyceae*) and the brown *Halopteris scoparia* (*Phaeophyceae*), sampled in South Portugal. Bacterial enrichment cultures supplemented with chlorinated aliphatic compounds, typically released by marine algae, were established using as inoculum the decaying biomass of the three macroalgae, obtaining a collection of 634 bacterial strains. Although collected from the same site and exposed to the same seawater seeding microbiota, macroalgal cultivable bacterial communities in terms of functional and phylogenetic diversity showed host specificity. Isolates were tested for the hydrolysis of starch, pectin, alginate and agar, exhibiting a different hydrolytic potential according to their host: *A. taxiformis* showed the highest percentage of active isolates (91%), followed by *S. coronopifolius* (54%) and *H. scoparia* (46%). Only 30% of the isolates were able to degrade starch, while the other polymers were degraded by 55–58% of the isolates. Interestingly, several isolates showed promiscuous capacities to hydrolyze more than one polysaccharide. The isolate functional fingerprint was statistically correlated to bacterial phylogeny, host species and enrichment medium. In conclusion, this work depicts macroalgae as holobionts with an associated microbiota of interest for blue biotechnologies, suggesting isolation strategies and bacterial targets for polysaccharidases’ discovery.

**Supplementary Information:**

The online version contains supplementary material available at 10.1186/s13568-022-01440-8.

## Keypoints

- Macroalgae are source of bacterial polysaccharidases for blue biotechnologies.
- Amilases, pectinases, alginate lyases and agarases were widespread among isolates.
- Hydrolytic profile correlates to strain phylogeny, host species, isolation medium.

**Supplementary Information:**

The online version contains supplementary material available at 10.1186/s13568-022-01440-8.

## Introduction

Marine environments, characterized by specific physicochemical parameters as well as by the presence of unusual carbon sources (Rao et al. 2017; Zeaiter et al. 2019), host organisms constituting a reservoir of biotechnologically relevant functions that could be exploited in medical, pharmaceutical and industrial applications (Iwamoto et al. 2001; Wang et al. 2020; Rodrigues et al. 2017).

In the seawater milieu, surface of macroalgae constitutes a particular ecological niche and an advantageous substratum for microorganisms, which rely for their nutritional needs on the organic substances secreted by their host (Michel et al. 2006). Albeit it was suggested that common members of algal bacterial communities constitute a core group (Selvarajan et al. 2019), the specific recruitment of bacteria on algal surfaces depends on defined functional traits, and is regulated by the algal host itself (Egan et al. 2013; Behringer et al. 2018; Crenn et al. 2018). Some algal species are characterized by distinct associated bacterial communities, in relation to the composition of algal surfaces and exudates (Kimbrel et al. 2019), sampling season (Lachnit et al. 2011), as well as geographic location (Singh and Reddy 2014). A long evolutionary history of cross-kingdom interactions between algae and epiphytic bacteria has therefore shaped complex associations and specializations between the two counterparts, which contribute to host health, morphological development and defense and allow to define algae and their associated microbes as holobionts, as analogously described for corals (Rosenberg et al. 2007). In particular, epiphytic bacteria provide assistance with metabolic processes, i.e., nitrification, nitrogen fixation, sulfate reduction, photosynthesis, nutrient exchange, plant growth enhancement, quorum sensing mediation, host chemical defense via antifouling properties against unwanted colonization and episodic predatory behaviors (Egan et al. 2008, 2013; Singh and Reddy 2014; Barott et al. 2011; Burke et al. 2011; Cirri and Pohnert 2019). On the other hand, an array of biological, physical, and chemical properties of macroalgal surfaces is probably involved in structuring the associated epiphytic microbial community and its metabolic activity (Egan et al. 2013). Therefore, understanding macroalgae functioning from an ecological perspective cannot overlook the interactions with their associated microbiome.

Polysaccharide-degrading bacteria are important members of the macroalgal bacterial community: by using algal cell wall polymers as carbon source, they contribute to the global carbon cycle (Gupta et al. 2013) and, broadly, to macroalgal holobiont maintenance (Martin et al. 2015). Some bacterial species can play a crucial role when macroalgal degradation processes occur, allowing the decay of macroalgae with consequent carbon compound recycling in the marine environment (Imran et al. 2017). It is therefore of particular interest to focus attention on the specific microbial community that dominates the surface of decaying, besides than healthy, macroalgae (Ihua et al. 2019).

Macroalgal cell wall composition is characterized by sulfated polysaccharides, not present in plants (Popper et al. 2011). Among cell wall constituents there are few carbohydrates of lignocellulosic nature (hexoses-glucose, mannose and galactose, pentoses-xylose and arabinose) and a large array of polysaccharides (e.g., agar, carrageenan, alginate, fucan, laminarin, cellulose, and pectin). A wide diversity of glycans, as cell wall components and energy storage compounds, is produced by macroalgae and could have potential applications in industrial waste and by-products treatments. Specifically, while red algae are characterized by a cell wall mainly composed of cellulose, sulfated galactans, mannan, xylan, carrageenan and agar, polysaccharides found in brown macroalgae are largely represented by alginate, fucoidan, laminarin and cellulose (Popper et al. 2011). Taking advantage of high productivity, high content of carbohydrates and lipids, and the possibility of bulk-scale farming without the need for fertilizers (Ferdouse et al. 2018), macroalgae cultivation is increasing in Europe, meeting the request of industry for sustainable biomass resources together with the emerging awareness for sustainability in food production (Wang et al. 2020). Under this perspective the identification of enzymes able to degrade algal components is of paramount importance for many biotechnological applications.

The majority of the degrading enzymes targeting macroalgal polysaccharides (polysaccharide lyases and glycoside hydrolases, Michel and Czjzek 2013) have been isolated from macroalgae-associated bacteria belonging to *Gammaproteobacteria* (phylum *Proteobacteria)* and *Flavobacteria* (phylum *Bacteroidetes*) classes (Nedashkovskaya et al. 2014; Martin et al. 2014). Knowledge is nevertheless still scarce regarding the enzymes produced by the macroalgae-associated epibiota. Aim of this work was to isolate algalytic bacteria from industrial interesting macroalgae, growing in sympatry but with a contrasting phylogenetic background, and to link the polysaccharidase activity of the isolates with their phylogeny, host species and enrichment medium, thereby aiming to identify the most promising putative target bacterial genera and isolation procedures to mine novel polysaccharide degrading enzymes from the marine habitat.

## Materials and methods

### Enrichment media and bacterial isolation

*Asparagopsis taxiformis*, *Halopteris scoparia* and *Sphaerococcus coronopifolius* specimens were collected in November 2018, by snorkeling on the coast of South Portugal. Samples, maintained in the local seawater, were delivered at the University of Milan stored on ice then and incubated intact for 30 days at 4 °C to induce a controlled natural biomass decay process, prior to be processed for bacteria enrichment and isolation. Macroalgae were firstly rinsed with sterile milliQ water to remove loosely attached bacteria, then 2.5 g of sample was used as inoculum in 250 mL artificial seawater (ASW, sterilized by filtration with 0.22 μm pore filters) added with 100 mg/L of either 1,2-dichloroetane (1,2-DCA) or 1,2-dibromoethene (1,2-DBE) and incubated at 30 °C under mild rotating agitation. Medium turbidity appeared 3 days after microcosms’ establishment, after which 2.5 mL of the supernatant was transferred, avoiding visible algal original biomass, in 250 mL of the same fresh medium. This procedure was repeated four times and subsequently the bacterial suspension was plated on ONR7a agarized medium (DSMZ medium 950) with the addition of either 1,2-DCA or 1,2-DBE, and incubated at 30 °C until colony appearance (5–7 days). Pure cultures were obtained by streaking isolates three times on the same medium. Both enrichment and isolation media were added with 100 mg/L of cycloheximide to inhibit fungal growth. Cultures from the first inoculum were also plated on agarized Marin Broth (MA) (Conda), incubated at 30 °C until colonies appeared (1–3 days) and streaked at single colony three times. A graphical scheme of the adopted isolation procedure is reported in Additional file 1: Fig. S1. Chemicals, except were specifically reported, were purchased from Sigma-Aldrich.

### Identification of bacteria associated with the macroalgae

DNA was extracted from a single colony of each isolate through a boiling lysis procedure (Ferjani et al. 2015). Bacterial collection was dereplicated by intergenic internal transcribed spacer (ITS)-PCR fingerprinting, using the primers ITS-F (3′-GTCGTAACAAGGTAGCCGTA-5′) and ITS-R (3′-CTACGGCTACCTTGTTACGA-5′), as previously described (Barbato et al. 2016). At least one representative for each ITS group was identified by partial 16 S rRNA sequencing and subsequent alignment of the sequence in NCBI database (http://www.ncbi.nlm.nih.gov/BLAST/Blast.cgi). The amplification of the bacterial 16 S rRNA gene was performed using the universal primers 27 F (3′-AGAGTTTGATCMTGGCTCAG-5′) and 1492R (3′-CTACGGCTACCTTGTTACGA-5′) (Mapelli et al. 2013). Partial 16 S rRNA gene sequences obtained from the bacterial isolates are available at the European Nucleotide Archive (ENA) under the study accession numbers PRJEB43423 (HG994980-HG995020 and HG999366-HG999678).

### Screening of the bacterial hydrolytic profile

All the isolates were tested for extracellular polysaccharide-degradation on starch, pectin, alginate and agar following a protocol modified from Jain and Krishnan (2017). Briefly, 10 µL of an overnight liquid culture of each isolate were spotted on agar plates containing either MA or ONR7a modified media supplemented with starch (0.5% w/V), alginate (0.2% w/V) and pectin (0.2% w/V). Plates solidified using agar (1.5% w/V) were used for agarolytic activity. Prior autoclaving, the pH of the growth media was adjusted to 7.2 with the addition of NaOH. After inoculation, the plates were incubated at 30 °C for 3 days. To observe the degradation halo for each polysaccharide, the plates were flooded with the appropriate reagent for 5 min and rinsed. Lugol’s iodine reagent was used to detect agarolytic isolates (Mai et al. 2016), while Gram’s Iodine was used to detect hydrolysis of the other polysaccharides (Sunnotel and Nigam 2002; Sawant et al. 2015). The qualitative extent of the degrading activity was expressed as the ratio between degradation halo diameter (mm) and bacterial colony diameter (mm), allowing the comparison among isolates.

### Statistical analyses

Statistical analyses were performed with the software PRIMER7 (Clarke and Gorley 2015), PERMANOVA + for PRIMER routines (Anderson et al. 2008) and with Calc Statistical Function of Microsoft^R^ Office (Student’s t test). A dissimilarity matrix was built basing on results of the tested activities for each isolate and similarities among isolates’ activities according to bacterial taxonomy and macroalgal hosts were investigated by non-metric multi-dimensional scaling (nMDS). Significant differences of the hydrolytic profiles in the bacterial collection (including the level of the enzymatic activity) were investigated through Permutational Multivariate Analysis of Variance (PERMANOVA) and Monte Carlo P test considering the bacterial taxonomy, growth media and macroalgal hosts. Distribution of hydrolytic activities in the three algal sub-collections, as well as considering the halogenated compounds and isolation media, was analyzed with Calc Statistical Function of Microsoft^R^ Office applying Student’s t test.

## Results

### Bacterial collections obtained from the different macroalgae

A total of 634 bacterial isolates were obtained as pure cultures from the three algal species: 221 isolates from *A. taxiformis*, 194 from *H.* scoparia and 219 from *S. coronopifolius*. We considered that most of the obtained isolates were epiphytes, but we could not exclude that endophytic bacteria could be present, derived from decaying macroalgal material (Ihua et al. 2019). The bacterial isolates were clustered in 287 groups based on their ITS profiles and the partial 16 S rRNA gene of at least one representative for each ITS group was sequenced for identification purposes (see "Materials and methods" section for the sequence accession numbers; Additional file 2: Table S1).

**Fig. 1:**
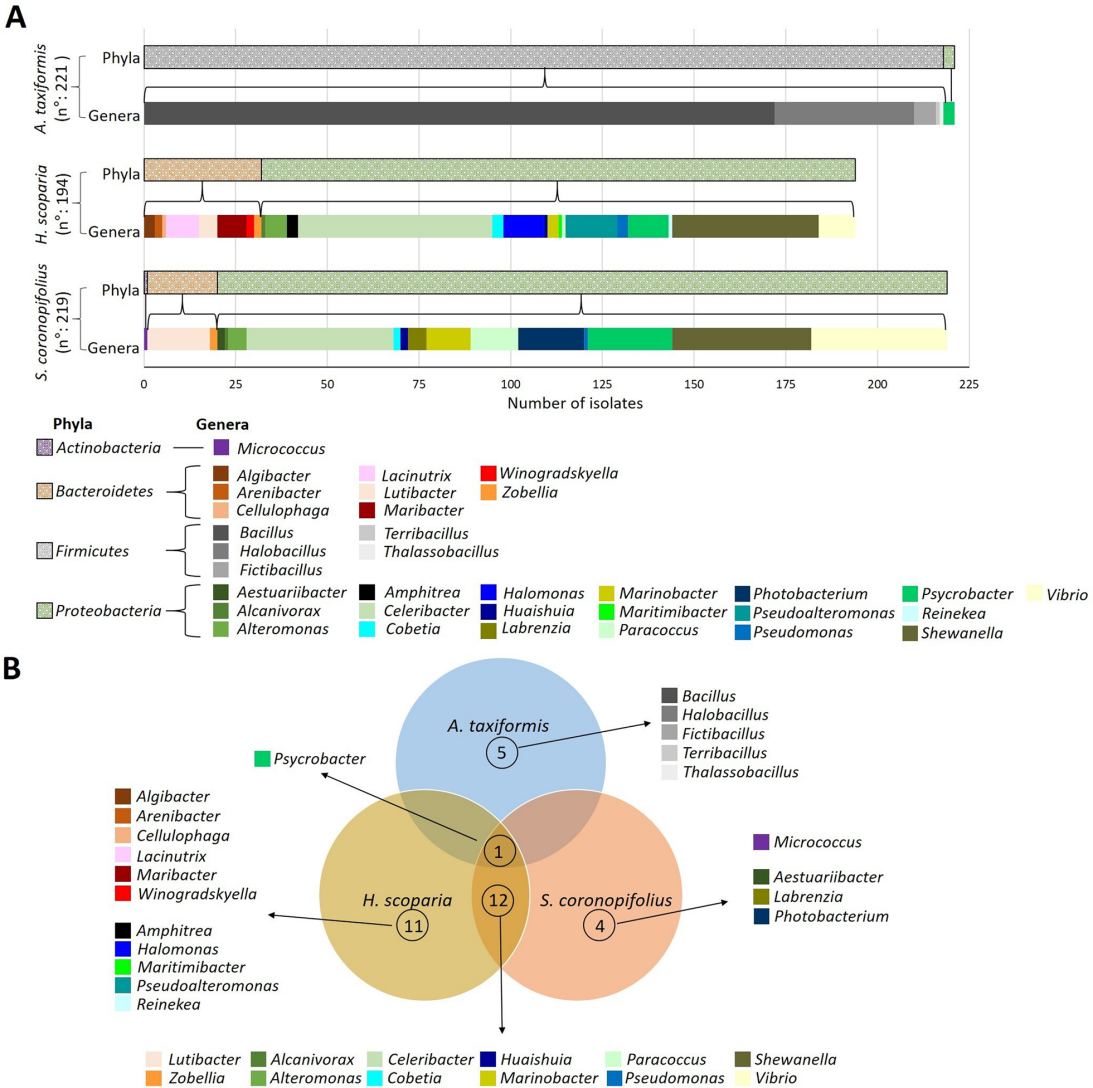
Bacterial collections. **A** Taxonomic composition of bacterial isolates at phyla and genera levels for each macroalga: *Asparagopsis taxiformis*, *Sphaerococcus coronopifolius* (both Rhodophycea) and the brown *Halopteris scoparia* (*Phaeophyceae*). **B** Venn diagram showing the bacterial genera unique for each or shared among the three macroalgae

The three bacterial collections spanned 33 different genera belonging to 3 phyla: *Bacteroidetes* (51 isolates, 8% of the overall collection), *Firmicutes* (218 isolates, 34% of the overall collection) and *Proteobacteria* (364 isolates, 57% of the overall collection). Only one isolate belonged to the phylum *Actinobacteria*, *Micrococcus luteus*, and was obtained from *S. coronopifolius* (Fig. 1A, Additional file 2: Table S1).

The taxonomic composition of the isolate collections differed among hosts, regardless host phylogenetic relationship. Members of the *Bacteroidetes* phylum were retrieved mainly from *H. scoparia*, *Phaeophyceae*, (32 isolates) and *S. coronopifolius*, *Rhodophyceae*, (19 isolates), whereas *Firmicutes* were isolated exclusively from *A. taxiformis, Rhodophyceae*. *Proteobacteria* represented more than half of the overall collection and were retrieved from all three algae with the highest prevalence in *S. coronopifolius* (199 isolates, 91% of the collection isolated from this host), *H. scoparia* (162 isolates, 84% of the collection isolated from this host), and only 3 isolates isolated from *A. taxiformis* (1% of the collection). These latter isolates belonged to the *Psychrobacter* genus (Fig. 1A, Additional file 2: Table S1), which resulted the only common genus among the 3 collections (Fig. 1B). Thirteen bacterial genera were shared between *H. scoparia* and *S. coronopifolius* (Fig. 1A, B, Additional file 2: Table S1).

### Bacterial isolate hydrolytic activities on starch, pectin, alginate and agar

**Fig. 2:**
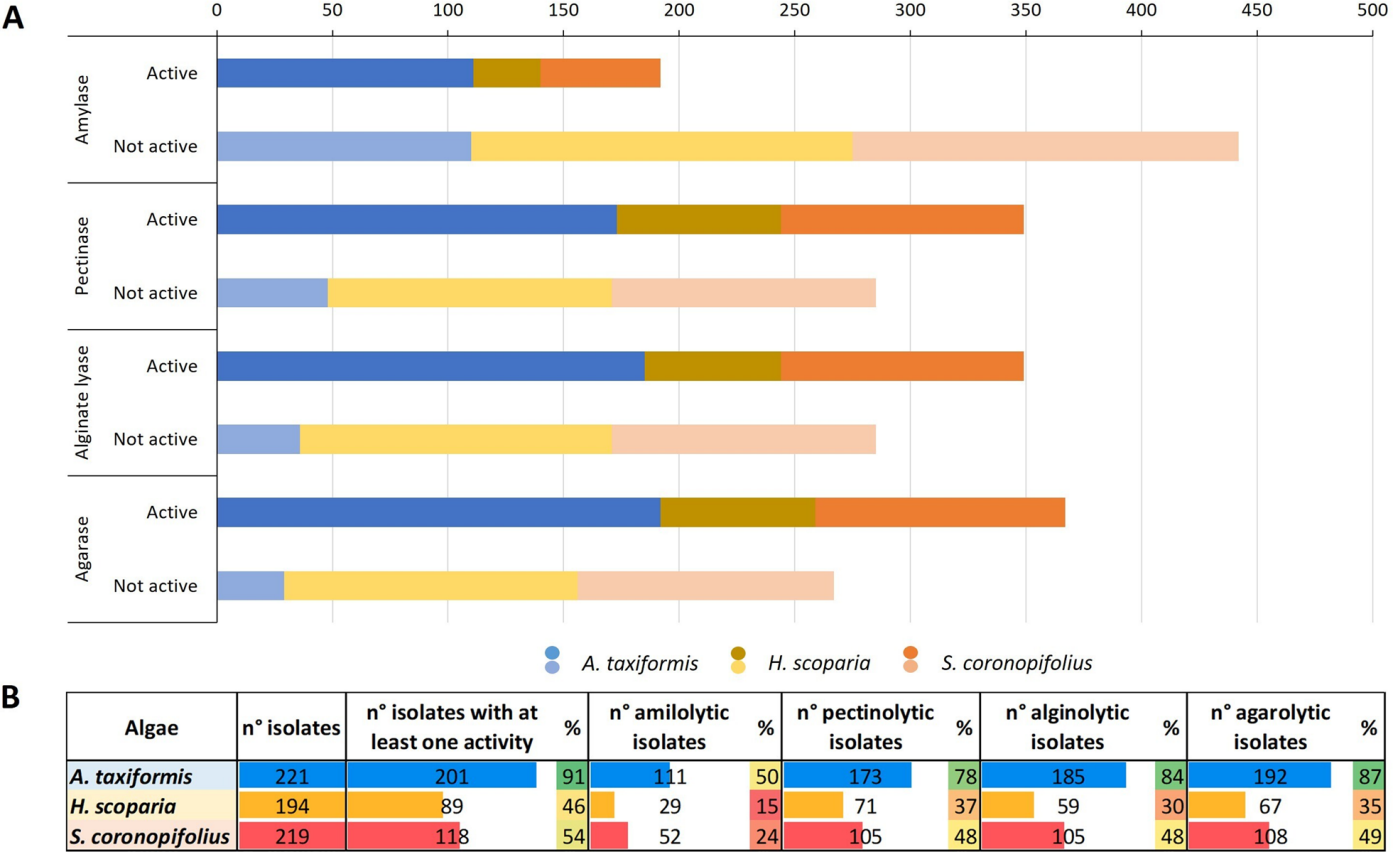
Hydrolytic activities of bacteria isolated from the macroalgae *Asparagopsis taxiformis*, *Halopteris scoparia* and *Sphaerococcus coronopifolius* on starch, pectine, alginate and agar. **A** Active (dark colors) and not-active (light colors) isolates considering the 3 macroalgal species and the different hydrolytic activities. **B** Number of active isolates for each macroalgal species. Percentages represent the fraction of active isolates in each algal sub-collection. Colors in the percentage boxes are related to the percentage values: higher percentages are reported in green, intermediate percentages in yellow and lower percentages are in red. Bars represent the numbers of active isolates

**Fig. 3:**
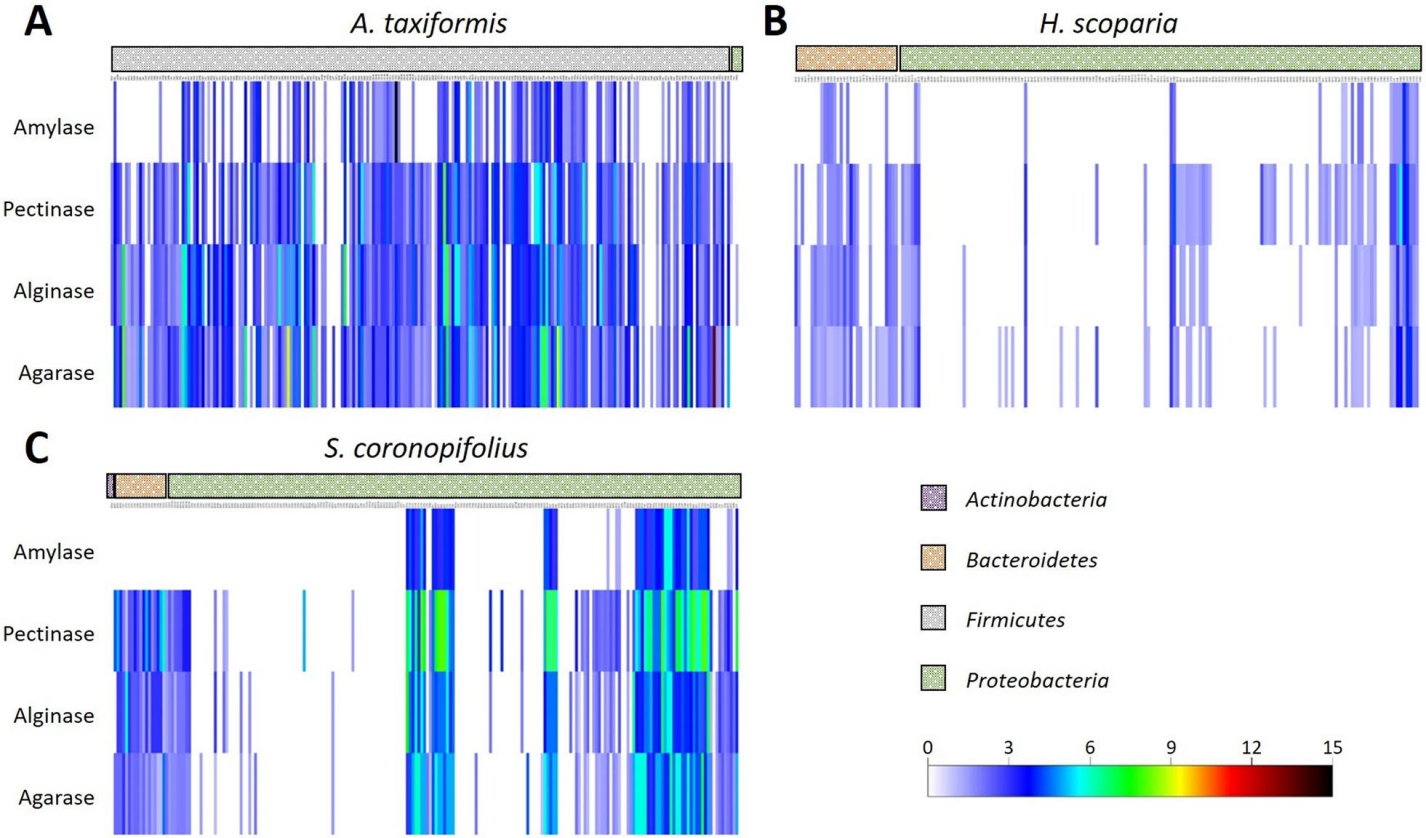
Heat map of the tested activities (amylase, pectinase, alginate lyase, agarase) for each isolate in the macroalgae: **A** *Asparagopsis. taxiformis*; **B** *Halopteris scoparia*; and **C** *Sphaerococcus coronopifolius.* Colors indicate the activity level calculated as the ratio between the degradation halo diameter and the isolate growth diameter

Isolates were tested to uncover the potential of the epiphytic macroalgae bacterial community as a source of degrading enzymes on starch, pectin, alginate and agar polysaccharides. Detailed results of the activity tests are reported in Additional file 2: Table S2. The bacterial collection established from *A. taxiformis* showed the highest percentage of isolates (91%) showing $\ge 1$ polysaccharidase activity, followed by the *S. coronopifolius* and *H. scoparia* collections, with 54 and 46%active isolates, respectively (Fig. 2A, B; Additional file 2: Table S2). Considering the quali-quantitative evaluation of the degrading activity, estimated from the size of the hydrolysis haloes on agar plate cultures, the *H. scoparia* collection demonstrated also the lower levels of activity when compared with the collections obtained from two red macroalgae (Fig. 3).

The polysaccharidase activity was widespread in the collections: 30% of the isolates showed degradation of starch, 55% pectin, 55% alginate, and 58% agar. The relative abundance of active isolates differed among the collections obtained from the three hosts (Figs. 2B and 3A–C, Additional file 2: Table S2). Isolate BP26, the only representative of the *Actinobacteria* phylum in the whole collection, did not show any hydrolytic activity, whereas we found representatives with at least one activity in the other three identified phyla (*Proteobacteria* with the classes *Alphaproteobacteria* and *Gammaproteobacteria*; *Firmicutes* with the class *Bacilli* and *Bacteroidetes* with the class *Flavobacteriia*) (Fig. 3).

91% of the *Firmicutes*, isolated uniquely from *A. taxiformis*, demonstrated polysaccharidase activity: the capacity to degrade all the tested polysaccharides was widespread among isolates belonging to 4 Firmicutes genera (*Bacillus*, *Fictibacillus*, *Halobacillus* and *Thalassobacillus*; Table 1). Among *Proteobacteria*, mainly obtained from *H. scoparia* and *S. coronopifolius*, isolates belonging to 12 genera demonstrated polysaccharidase activity. Only six genera displayed amylolytic capacity, while hydrolyses of the other polysaccharides were more widespread (isolates belonging to 11, 11 and 12 genera showed, respectively, pectinase, alginase and agarase activity). Among the 37 *Psychrobacter* sp. isolates, the unique genus isolated from all the algae, none exhibited amylase activity, while only 6 possessed at least one hydrolytic activity. 90% of the *Bacteroidetes* isolates were active in polysaccharide degradation (with at least one hydrolytic activity): only a few number of genera (n = 3) showed to be amylolytic, while 7 genera were active on the other polysaccharides (Additional file 1: Fig. S2).

**Table 1:** Overview of active algalytic bacterial isolates obtained from the macroalgae *Asparagopsis taxiformis*, *Halopteris scoparia* and *Sphaerococcus coronopifolius*

	Phyla	n°	Genera	n°	Macroalgae		
					*A. taxiformis*	*H. scoparia*	*S. coronopifolius*
Amylase activity	*Proteobacteria*	73	*Alteromonas*	2	–	2	–
192 active isolates			*Celeribacter*	1	–	1	–
			*Photobacterium*	15	–	–	15
			*Pseudoalteromonas*	2	–	2	–
			*Shewanella*	15	–	7	8
			*Vibrio*	38	–	9	29
	*Firmicutes*	111	*Bacillus*	88	88	–	–
			*Fictibacillus*	5	5	–	–
			*Halobacillus*	17	17	–	–
			*Thalassobacillus*	1	1	–	–
	*Bacteroidetes*	8	*Lacinutrix*	6	–	6	–
			*Lutibacter*	1	–	1	–
			*Winogradskiella*	1	–	1	–
	Total number of active isolates				111	29	52
Pectinase activity	*Proteobacteria*	140	*Aestuariibacter*	2	–	–	2
349 active isolates			*Alcanivorax*	1	–	–	1
			*Alteromonas*	11	–	6	5
			*Celeribacter*	6	–	2	4
			*Halomonas*	1	–	1	–
			*Marinobacter*	1	–	–	1
			*Photobacterium*	16	–	–	16
			*Pseudoalteromonas*	13	–	13	–
			*Psychrobacter*	4	1	–	3
			*Shewanella*	42	–	22	20
			*Vibrio*	43	–	9	34
	*Firmicutes*	172	*Bacillus*	138	138	–	–
			*Fictibacillus*	6	6	–	–
			*Halobacillus*	27	27	–	–
			*Thalassobacillus*	1	1	–	–
	*Bacteroidetes*	37	*Algibacter*	2	–	2	–
			*Lacinutrix*	7	–	7	–
			*Lutibacter*	21	–	4	17
			*Maribacter*	1	–	1	–
			*Winogradskiella*	2	–	2	–
			*Zobellia*	4	–	2	2
	Total number of active isolates				173	71	105
Alginase activity	*Proteobacteria*	125	*Aestuariibacter*	2	–	–	2
349 active isolates			*Alcanivorax*	1	–	–	1
			*Alteromonas*	11	–	6	5
			*Celeribacter*	7	–	2	5
			*Halomonas*	1	–	1	–
			*Marinobacter*	1	–	–	1
			*Photobacterium*	16	–	–	16
			*Pseudoalteromonas*	9	–	9	–
			*Psychrobacter*	3	1	–	2
			*Shewanella*	29	–	10	19
			*Vibrio*	45	–	9	36
	*Firmicutes*	184	*Bacillus*	150	150	*–*	*–*
			*Fictibacillus*	6	6	–	–
			*Halobacillus*	27	27	–	–
			*Thalassobacillus*	1	1	–	–
	*Bacteroidetes*	40	*Algibacter*	2	–	2	–
			*Cellulophaga*	1	–	1	–
			*Lacinutrix*	9	–	9	–
			*Lutibacter*	21	–	5	16
			*Maribacter*	1	–	1	–
			*Winogradskiella*	2	–	2	–
			*Zobellia*	4	–	2	2
	Total number of active isolates				185	59	105
Agarase activity	*Proteobacteria*	131	*Aestuariibacter*	2	–	–	2
367 active isolates			*Alcanivorax*	1	–	–	1
			*Alteromonas*	11	–	6	5
			*Celeribacter*	13	–	8	5
			*Halomonas*	1	–	1	–
			*Huaishuia*	1	–	1	–
			*Marinobacter*	1	–	–	1
			*Photobacterium*	17	–	–	17
			*Pseudoalteromonas*	8	–	8	–
			*Psychrobacter*	1	–	–	1
			*Shewanella*	30	–	9	21
			*Vibrio*	45	–	9	36
	*Firmicutes*	192	*Bacillus*	155	155	–	–
			*Fictibacillus*	6	6	–	–
			*Halobacillus*	30	30	–	–
			*Thalassobacillus*	1	1	–	–
	*Bacteroidetes*	44	*Algibacter*	2	–	2	–
			*Cellulophaga*	1	–	1	–
			*Lacinutrix*	9	–	9	–
			*Lutibacter*	21	–	4	17
			*Maribacter*	5	–	5	–
			*Winogradskiella*	2	–	2	–
			*Zobellia*	4	–	2	2
	Total number of active isolates				192	67	108

**Fig. 4:**
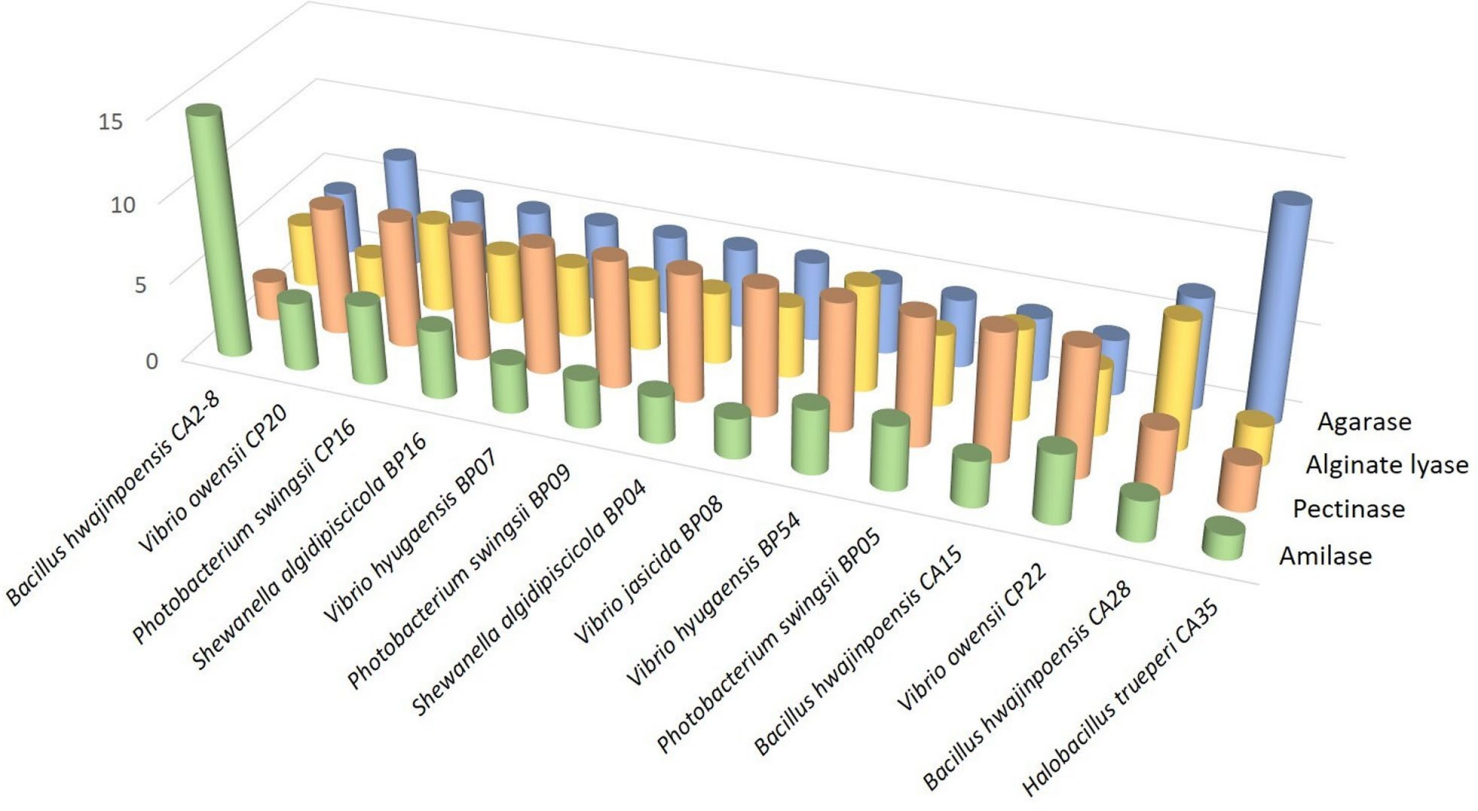
Activity levels (reported as the ratio between the degradation halo diameter and the isolate growth diameter) of the bacterial isolates from the macroalgae *Asparagopsis taxiformis*, *Halopteris scoparia* and *Sphaerococcus coronopifolius* with the highest activities. Specifically, isolate CA2-8 showed the highest amylase activity (value 15), while 11 isolates showed the highest activity on pectin (value 8); isolate CA28 had the highest alginate lyase activity (value 8) and isolate CA35 showed the highest measured agarase activity (value 13)

*Bacillus hwajinpoensis* CA2-8 showed the highest amylase activity (with a value of 15, calculated as the ratio between the degradation halo diameter and the isolate growth diameter), at least five times higher than the other isolates (Additional file 2: Table S3, Fig. 4). Interestingly, another isolate belonging to the same species, i.e.,* Bacillus hwajinpoensis* CA28, showed the highest alginate lyase activity (value 8). The isolate *Halobacillus trueperi* CA35 showed the highest agarase activity (value 13), whereas 11 isolates displayed the highest pectinase activity (value 8): one *Firmicutes* (*Bacillus hwajinpoensis* CA15), and 10 *Proteobacteria* (Table 1; Additional file 2: Table S3; Fig. 4).

The functional potential of the collection was often correlated with the isolate phylogeny (Additional file 2: Table S4). Isolates were randomly selected, thus taxonomical groups were represented unbalanced: to evaluate the genera with the most interesting activity, we considered only those represented by at least 15 isolates (Additional file 2: Table S4; taxa highlighted in violet). *Vibrio* and *Photobacterium* (*Vibrionaceae* family) included isolates active on the different polysaccharides with high percentages of activity, ranging between 81 and 96%. Conversely, *Marinobacter* and *Psychrobacter* (*Gammaproteobacteria*) and *Celeribacter* (*Firmicutes*) comprised isolates with low activity. *Lutibacter*, *Bacillus*, *Halobacillus* and *Shewanella* isolates showed to be more active on pectin, alginate and agar, than on starch. Interestingly, if a genus showed amylase activity, it also possessed the other 3 hydrolytic activities (Additional file 2: Table S4).

Finally, considering the active genera shared between *H. scoparia* and *S. coronopifolius*, only *Shewanella* and *Vibrio* isolates were active on starch, while isolates belonging to a larger number of genera were active on the other tested polysaccharides: *Alteromonas*, *Lutibacter*, *Zobellia*, *Celeribacter, Shewanella* and *Vibrio* (Table 1; Additional file 2: Table S4).

### Correlation between strain phylogeny, isolation strategy and functional potential

We investigated the correlation between the isolates’ hydrolytic fingerprint and: (i) the taxonomical identification of the strain, (ii) the macroalgal source, and (iii) the enrichment/isolation conditions, considering for each isolate both the polysaccharide(s) hydrolyzed and the specific level of activity. Aim of the analyses was to evaluate which factor(s) could be considered putatively responsible for obtaining isolates with a specific degrading activity. Considering the whole bacterial collection, the hydrolytic fingerprint of the isolates differed in relation to their taxonomical affiliation, algal source and isolation medium (Additional file 2: Table S5A, B).

Considering that algal source and strain taxonomical affiliation are not independent factors (Aires et al. 2016), PERMANOVA analysis was applied also considering separately the three bacterial sub-collections obtained from the different macroalgae, only for the taxonomical groups including at least 6 isolates (Additional file 1: Fig. S2; Additional file 2: Tables S6–S8). Within *A. taxiformis* collection it was possible to observe differences in polysaccharidase distribution according to the strain phylogenetic affiliation at the genus level (Additional file 2: Table S6A): the hydrolytic properties showed by *Halobacillus* strains significantly differed from those of the *Bacillus* and *Fictibacillus* genera (Additional file 2: Table S6B). Moreover, the hydrolytic fingerprint differed considering the halogenated hydrocarbons added to the enrichment media (1,2-DBE or 1,2-DCA), but not the basal isolation medium (ONR7a or MB) (Additional file 2: Table S6A). Within *H. scoparia* collection, the isolate degrading fingerprint differed based on the basal isolation medium, the added halogenated hydrocarbon and the taxonomic level (Additional file 2: Table S7A, B). At the order level, we did not observe significant differences among *Oceanospirillales*, *Rhodobacterales* and *Pseudomonadales* isolates (Additional file 2: Table S7C). The degrading profile of the isolates obtained from *S. coronopifolius* was significantly different based on the basal isolation medium and strain taxonomical affiliation (except for phyla level), but not on the added halogenated substrate (Additional file 2: Table S8A). Nonetheless, when pairwise comparisons were performed, a significant difference was highlighted between *Bacteroidetes* and *Proteobacteria* (Additional file 2: Table S8B).

**Fig. 5:**
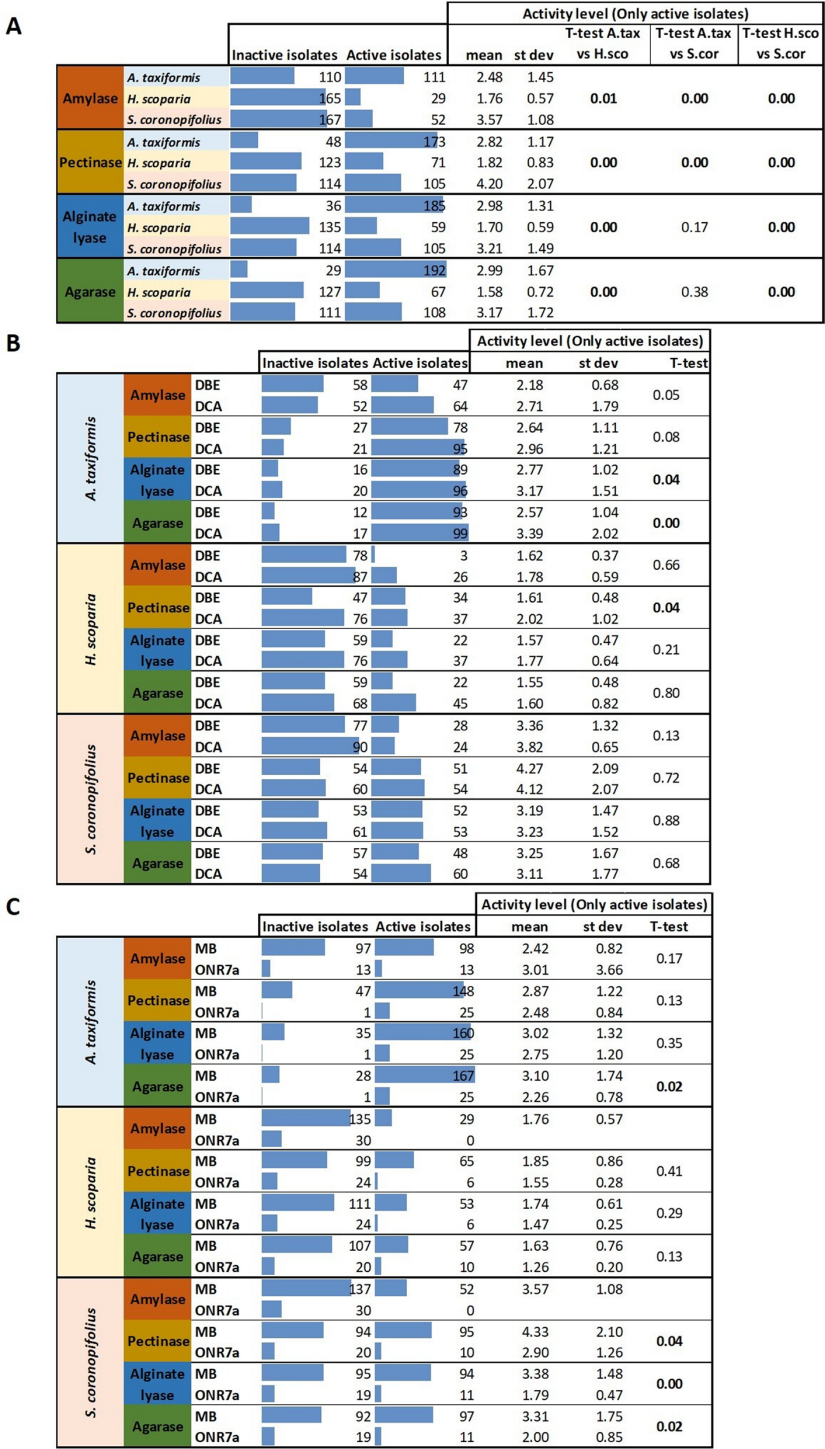
Distribution of hydrolytic activities (i.e., amylase, pectinase, alginate lyase and agarase) in the 3 macroalgal sub-collections (**A**), and considering the halogenated compounds (1,2-DBE vs. 1,2-DCA, **B**) and isolation media (MB vs. ONR7a, **C**). Statistical analysis (Student’s t-test) was conducted with Calc Statistical Function of Microsoft^R^ Office. In bold: p < 0.05

To point out differences in relation to the polysaccharidases in terms of activity level, we analyzed their distribution in all the three algal sub-collections (Fig. 5A) and also considering the added halogenated compounds (Fig. 5B) and the basal isolation media (Fig. 5C).

Higher levels of amylase and pectinase activities were obtained by isolates originating from *S. coronopifolius* than from the other two macroalgae, while alginate lyase and agarase activities were high both among *A. taxiformis* and *S. coronopifolius* isolates (Fig. 5A).

1,2-DCA-supplemented enrichments compared with 1,2-DBE-supplemented media increased the number of alginate lyase and agarase positive isolates in the *A. taxiformis* collection, and the number of pectinase positive isolates in the *H. scoparia* collection (Fig. 5B). Considering the basal isolation media, MB resulted in a higher number of agarase positive isolates in the *A. taxiformis* collection and a higher number of pectinase, alginate lyase and agarase positive isolates in the *S. coronopifolius* collection (Fig. 5C). However, it is noteworthy to underline that the result could be affected by the fact that a higher number of isolates was obtained from MB than from ONR7a basal medium (Additional file 1: Fig. S1).

## Discussion

Recent studies have reported that diverse bacteria can colonize nutrient-rich surfaces of macroalgae, establishing strong associations with them (Martin et al. 2015). High-throughput DNA sequencing has revealed that some taxa are common members of macroalgal bacterial communities, e.g., members of *Actinobacteria* and *Proteobacteria* phyla (for the latter with the *Gamma*- and *Alpha*- subgroups), even if no similarities have been detected at lower taxonomical levels starting from families, indicating the presence of host species-specific epiphytic communities (Florez et al. 2017; Hollants et al. 2013). Moreover, when the whole algal bacterial community was considered, host intraspecific differentiation across biogeographic regions has been shown, underlining the influence of the environmental conditions on the algal microbiota (Aires et al. 2016).

Previous works showed that *Oceanospirillaceae* and *Rhodobacteraceae* (*Proteobacteria* phylum), as well as *Flavobacteriaceae* (*Bacteroidetes* phylum) families constitute the culturable core members of the epibacterial community of the brown alga *Ascophyllum nodosum* (Martin et al. 2015). The genera *Pseudoalteromonas*, *Shewanella*, and *Zobellia* have been identified within the culturable communities of red, brown and green macroalgae (Hollants et al. 2013). Although only a small proportion of the microbial strains associated to a specific host or habitat is cultivable, microbial cultivation is the gold standard to experimentally validate the presence of specific microbial metabolic capabilities and, consequently, to exploit the microbial potential for industrial applications (Prakash et al. 2013). Hence, with the final goal to setup specific and tailored isolation and cultivation conditions to mine novel hydrolytic enzymes of interest for biotechnological application, this study was based on a culture-dependent approach.

The 634 isolate collection of decaying macroalgae-associated bacteria included members belonging to 3 main phyla, partially overlapping with the taxa described in the abovementioned studies (i.e., *Bacteroidetes*, *Firmicutes* and *Proteobacteria*; Martin et al. 2015; Florez et al. 2017; Hollants et al. 2013; Aires et al. 2015). Moreover, our results provide insights in the role of the host species in defining the composition of the culturable bacterial communities: although the macroalgal specimen occurred in sympatry, thus exposed to the same seawater seeding bacterial community, the phylum- and genus- level taxonomical differences among the collections indicated that each macroalga constitutes a peculiar ecological microbial niche, able to exert a strong and species-specific selection pressure on the bacteria. These holobiont-related interactions confirm the results obtained by other studies performed on macroalgal species growing in sympatry, with epiphytic bacterial communities different in composition and abundance (Lachnit et al. 2009; Nylund et al. 2010). Burke and colleagues (2011) also reported the evidence of a high variability in the composition of bacterial communities even among members of the same species, although similar functional composition has been depicted for the bacteria through metagenomics analyses. Our results were in agreement with this study and showed that, despite different taxonomical composition of the epiphytic community, all the considered activities were represented in each macroalgae host, provided by various and different bacterial members (Fig. 2A; Table 1). It is worth to highlight that all activities were represented in the bacterial communities associated to the threee macroalgae by a considerable number of isolates, most of which generalists, i.e., active in the degradation of more than one polysaccharide.

Bacterial recruitment and specificity on algal surfaces rely on the selection of specific common functional traits (Crenn et al. 2018), which constitute a selective driving force. The lottery model proposed by Verster and Borenstein (2018) suggests a colonization by chance of epiphytic species, if equipped with the necessary functional traits, according to a sort of “first-come-first-served” principle. These traits may vary according to changes in site-specific environmental (Aires et al. 2016) and chemical conditions, e.g., pH, light availability, oxygen concentration, pollution and secondary metabolites (Burke et al. 2011; Campbell et al. 2015). Therefore, the settled community would not necessarily be phylogenetically related, instead it would constitute a so-called “functional guild” of members containing the suitable genes for the existing conditions (Roth-Schulze et al. 2016). It should be noted that, as a response to physiological and defense processes, macroalgae can indeed modify the surrounding environment. For example, changes and limitations in light and temperature in mesocosms’ trials have affected antifouling compounds’ production by the brown alga *Fucus vesiculosus*, with consequent modification of the composition and richness of its epiphytic microbial communities (Saha et al. 2014). Among the several compounds released, marine micro- and macroalgae (*Rhodophyceae* and *A*. *taxiformis* in particular; Kladi et al. 2004) produce halogenated metabolites, e.g., terpenes, halomethanes, phenols and short-chain hydrocarbons (Gschwend et al. 1985a, b; Paul and Pohnert 2011). In the present work, the use of 1,2-DCA and 1,2-DBE supplemented enrichment media was intended to mimic the algal surface environment, providing a strong selection factor for algae associated bacteria (Gschwend et al. 1985a, b). We choose, moreover, to increase this selection driver by using a much higher concentration of halogenated compounds than the physiological one with the aim to bring into culture novel microbial strains with potential industrial interest, able to survive in the harsh industrial conditions. Furthermore, we used as inoculum decaying macroalgae to enrich isolates with hydrolytic metabolism, responsible for carbon recycling in the marine milieu (Imran et al. 2017). Previous studies reported changes in macroalgal associated bacterial populations following conditions related to algal degradation (Ihua et al. 2019; Chun et al. 2017). The diversity of the bacterial community associated with the green alga *Cladophora* varied during decomposition, with a peculiar increase of genera adapted to increased ammonium-nitrogen levels (*Acinetobacter*, *Enterobacter*, *Kluyvera*, *Cedecea*) (Chun et al. 2017). A marked change in communities of the brown macroalga *Ascophyllum nodosum* upon decay was observed; in particular, a switch from *Bacteroidetes* to *Firmicutes* phyla in healthy and decaying tissues, respectively, and the presence of *Proteobacteria* in all the conditions tested was highlighted (Ihua et al. 2019). Interestingly, the authors reported that none of the isolates obtained from the intact macroalga showed polysaccharide-degrading activities, differently from the isolates retrieved from decaying samples (Ihua et al. 2019). In our work a lower number of isolates belonging to *Bacteroidetes* and *Actinobacteria* was recovered compared to *Firmicutes* and *Proteobacteria*. However, we adopted a different methodology than the work of Ihua and colleagues (2019): in the latter the decaying process was induced by incubation with higher temperatures and times, although no remarkable influence of temperature was reported on the isolate hydrolytic activity. In *A*. *nodosum*, the active isolates participating to the algal biomass recycling belonged to *Bacillus*, *Vibrio* and *Micrococcus* genera, which resulted in isolates capable of producing cell-wall degrading enzymes in conditions of limiting nutrients, e.g., decaying algae, in order to let algal polymers to be the primary source of nutrition (hydroxyethyl (HE)-cellulalse, lichenase, pectinase; Ihua et al. 2019).

The collection of polysaccharide-degrading (PD) bacteria that we established from decaying algae comprised more than 600 isolates, the majority of which (65%) positive for at least one of the tested hydrolyzing activities. This result is of considerable interest, in comparison to those reported by previous works aiming at the isolation of algae-associated PD bacteria. For example, Martin et al. (2015) obtained a collection of 324 bacterial isolates associated with the brown alga *Ascophyllum nodosum*, but solely the 24% of them showed PD activity. Sànchez Hinojosa and colleagues (Sánchez Hinojosa et al. 2018) found that only 12% of their 172 isolates, from three Antarctic macroalgae (*Himantothallus grandifolius*, *Phaeophyta*; *Himantothallus grandifolius* and *Plocamium cartilaginoum*, *Rhodophyta*), showed agarolytic activity. In contrast, a study performed on 207 bacterial isolates obtained from the green alga *Ulva lactuca*, 58% of the isolates produced amylases and/or agarases (Comba González et al. 2018). Low prevalence of PD isolates could depend on the utilization of healthy algal specimens, where the presence of bacteria capable of degrading cell wall components, dangerous for algal well-being and maintenance, is controlled and limited by the presence of other macroalga-associated bacteria (Hollants et al. 2013 and references therein). However, it is noteworthy that among the over 800 bacterial isolates obtained from decaying *A. nodosum* (brown macroalga) by Ihua et al. (2019), with a similar methodology as the one used here, only 7% showed PD activity. This difference with our results could be due to the type of activities tested since Ihua and colleagues (2019) tested HE-cellulase, lichenase and pectinase. This is confirmed by focusing only on pectinase activity (common in both studies), which resulted in a similar PD detection rate. In conclusion, differences in the prevalence of PD isolates obtained from healthy and decaying algae, as well as in different macroalgal species, highlight the importance of choosing the correct starting environmental conditions, specimens and isolation strategies when looking for specific enzymatic activities (Adam et al. 2018; Kato et al. 2018, 2020; Zheng et al. 2018; Barbato et al. 2019; Ishii et al. 2020), as also corroborated by the statistical analysis presented in this work. The use of different algal species and isolation conditions allowed to obtain a collection of bacterial isolates with different taxonomy and PD activity, which statistically correlated.

Within our collection, the genus *Bacillus* comprised the highest number of PD isolates, with the highest activity levels. *Bacillus* comprises well-known polysaccharidase producers and it has been estimated that about 50% of the enzymes commercially available are obtained from this genus (Schallmey et al. 2004). Isolates belonging to this genus could have an important role in the macroalgal decomposition process, also reported as the main PD genus isolated from decaying *(A) nodosum* (Ihua et al. 2019). Amylase activity is, in particular, well spread in this genus and *(B) amyloliquefaciens*, *B. licheniformis* and *B. stearothermophylus* are already exploited at the industrial level (Elyasi Far et al. 2020). In this work amylase was the PD activity detected with higher prevalence among the *Bacillus* isolates: in particular, *B. hwajinpoensis* CA2-8 showed an amylase activity five times higher than all the other tested isolates. *B. hwajinpoensis*, first isolated in 2004 from seawater (Yoon et al. 2004), has been proved to be able to degrade starch, but, to the best of our knowledge, no further investigations have been carried out regarding the amylase activity of this species. In addition, isolate CA2-8 showed degrading activity on all the other tested polysaccharides, indicating that it might be a good candidate for further investigations for industrial applications. Two isolates of *B. hwajinpoensis*, CA15 and CA28, showed also high pectinase and alginate lyase activity, respectively, further demonstrating the potential of this species. Pectinolytic activity of *Bacillus* strains has been well known since decades (Chesson 1980) and many studies reported *Bacillus* species, among which *Bacillus subtilis* in particular, as the best pectinase producers (Jayani et al. 2010; Rehman et al. 2012; Kavuthodi et al. 2015; Sohail et al. 2015; Kavuthodi and Sebastian 2018).

Alginate is one of the main components of seaweed cell wall, and alginate lyase activity of bacteria isolated from macroalgae has been already reported (Martin et al. 2015). Although alginate lyase has an important role in alginate degradation under mild condition, at present no efficient or specific enzymes are available to ease the industrial process of alginate monomerization. So far, few *Bacillus* isolates have been reported as good producers of alginate lyases (Chen et al. 2018; Wang et al. 2017), together with *Zobellia* spp. strains which are well known to degrade also other algal polysaccharides (Martin et al. 2014). Hence the isolation of novel strains having this activity is of particular interest for industrial biotechnology.

Together with alginate, agar is the other main constituent of seaweed cell wall and 58% of our isolates showed the capability to degrade it. Again, the highest number of isolates showing this activity were identified as *Bacillus* spp., even though the best performer, *Halobacillus trueperi* CA35, belonged to a related genus.

Among Gram negatives, *Shewanella* and *Vibrio* are the genera showing the highest numbers of PD isolates. S*hewanella algidipiscicola* isolates obtained from *S. coronopifolius* are among the best pectin degraders, together with *Photobacterium* and *Vibrio* isolates. *Shewanella* has a primary role in fish and seafood spoilage and *S. algidipiscicola* was first isolated from iced fish (Satomi et al. 2007), but its role in macroalgal decomposition has not yet been recorded. Previous works reported *Shewanella* capability to degrade pectin (Jain and Krishnan 2017) and starch (Yu et al. 2009; Selman et al. 2020) and two different novel alginate lyases were characterized in this genus (Yagi et al. 2018; Wang et al. 2015].

The observation that 177 isolates (28% of the overall collection) belonging to different genera such as *Bacillus*, *Vibrio*, *Halobacillus*, *Photobacterium* and *Shewanella*, showed degradation potential on all the different tested polysaccharide substrates might indicate their important role in macroalgal decomposition process and interesting potential for biotechnological exploitation. *Vibrio jashidia*, for example, has been previously proposed as a promising microorganism for macroalgal industrial pre-treatment to produce volatile fatty acids (Pham et al. 2013).

In conclusion, data here presented showed that decaying red and brown macroalgae can be a source of a high number of phylogenetically diverse cultivable epiphytic bacteria. Our results highlight the essential role played by the host algal species in defining the associated cultivable bacteria community and underlines the primary role of macroalgal species as ecological niche in shaping the associated microbiota. Moreover, this massive functional screening allowed to detect a remarkably high percentage of PD isolates belonging to divergent phylogenetic groups, several of which exhibiting substrate promiscuity. Statistical analyses demonstrated that, besides the original algal host, even the enrichment and isolation methods had a role in obtaining cultured strains with specific degrading activities targeting different macroalgal cell wall polysaccharides with potential for algae biomass exploitation. This work contributes to highlight that the epiphytic bacterial community associated to decaying macroalgae possesses a promising functional potential for blue biotechnologies (Ferrer et al. 2019). Future work should be devoted to investigate the novelty and/or relevance of the detected activities, specifically the related enzyme properties and their genetic determinants.

## Supplementary Information

**Additional file 1**: **FigureS1 **Schematic representation of the enrichment andisolation process of bacterial strains from decaying algae. The procedure wasapplied to the 3 different macroalgal species with the addition (separately) ofthe 2 different halogenated compounds (1,2-DBE and 1,2-DCA). ASW: artificialseawater. **FigureS2 **Non-metric MDS distribution of the isolates onthe basis of the tested polysaccharidase activities. Isolates are distinguishedon the basis of the **A**) alga, **B**) class, and type of activity, *i.e.***C**) amylase, **D**) pectinase, **E**) alginate lyase and **F**)agarase, considering the class level. In **C-F**) the size of the bubblesindicates the level of the enzymatic activity. Isolates negative for theactivity test are not represented.

**Additional file 2:** **Table S1.** Taxonomical identification of the isolates. The 16S rRNA gene for at least one representative of each ITS group was sequenced and taxonomic identification was attributed using Blastn against the NCBI public database. **Table S2.** Activity levels of all the isolates expressed as the ratio between the degradation halo diameter and the strain growth diameter. This table also contains information about the host species, the enrichment procedure, clustering in ITS groups and taxonomical identification of each isolate. **Table S3.** Activity levels of the strains showing the highest performance in at least one of the tested activities. Activity level is reported as the ratio between the degradation halo diameter and the strain growth diameter. Highest activity levels are highlighted in grey. **Table S4.** Summary of the functional potential of the collection on the basis of the isolate phylogeny. We reported the number of isolates (“N. Isol.”) belonging to each genus and the percentage of isolates active for each tested activity. In violet are highlighted the taxonomical groups that include more than 15 isolates. **Table S5.** PERMANOVA and Monte Carlo test performed on the whole isolate collection activity level. A) Main and B) pairwise comparisons. Pseudo F = pseudo-F ratio. P = permutation P-value. Unique perms = unique values of the test statistic. Pmc = P Monte Carlo value. Asterisk (*) indicates significant differences between samples (P < 0.05). **Table S6.** PERMANOVA and Monte Carlo test performed on A. taxiformis sub-collection activity level. A) Main test and B) pairwise comparisons. Pseudo F = pseudo-F ratio. P = permutation P-value. 3 Unique perms = unique values of the test statistic. Pmc = P Monte Carlo value. Asterisk (*) indicates significant differences between samples (P < 0.05). **Table S7.** PERMANOVA and Monte Carlo test performed on H. scoparia sub-collection activity level. A) Main test and B-C) pairwise comparisons according to B) classes and C) orders. Pseudo F = pseudo-F ratio. P = permutation P-value. Unique perms = unique values of the test statistic. Pmc = P Monte Carlo value. Asterisk (*) indicates significant differences between samples (P < 0.05). **Table S8.** PERMANOVA and Monte Carlo test performed on S. coronopifolius sub-collection activity level. A) Main test and B-C) pairwise comparisons according to B) phyla and C) orders. Pseudo F = pseudo-F ratio. P = permutation P-value. Unique perms = unique values of the test statistic. Pmc = P Monte Carlo value. Asterisk (*) indicates significant differences between samples (P < 0.05).

## Data Availability

All data generated or analyzed during this study are included in the manuscript and additional information.
