# Supplementary material for: What lies on macroalgal surface: diversity of polysaccharide degraders in culturable epiphytic bacteria
Source: AMB Express. 2022 Jul 27;12:98. doi: 10.1186/s13568-022-01440-8 (PMC9329506; doi:10.1186/s13568-022-01440-8)

Additional materials of

What lies on macroalgal surface: diversity of polysaccharide degraders in epiphytic bacterial communities

AMB Express

Marta Barbato[1,#], Violetta Vacchini[1], Aschwin H. Engelen[2], Giovanni Patania[1], Francesca Mapelli[1], Sara Borin[1,*], Elena Crotti[1]

[1]Dipartimento di Scienze per gli Alimenti, la Nutrizione e l'Ambiente (DeFENS), Università degli Studi di Milano, via Celoria 2, 20133 Milano, Italy

[2]Centro de Ciências do Mar (CCMAR), Universidade do Algarve, Faro, Portugal

[#]present address: Section of Microbiology, Department of Biology, Aarhus University, Aarhus, Denmark.

*Corresponding author at Dipartimento di Scienze per gli Alimenti, la Nutrizione e l'Ambiente (DeFENS), Università degli Studi di Milano, via Celoria 2, 20133 Milano, Italy. Phone: +39.02.50319118. Fax: +39.02.50319238. Email address: sara.borin@unimi.it

Figures

Figure S1 Schematic representation of the enrichment and isolation process of bacterial strains from decaying algae. The procedure was applied to the 3 different macroalgal species with the addition (separately) of the 2 different halogenated compounds (1,2-DBE and 1,2-DCA). ASW: artificial seawater.

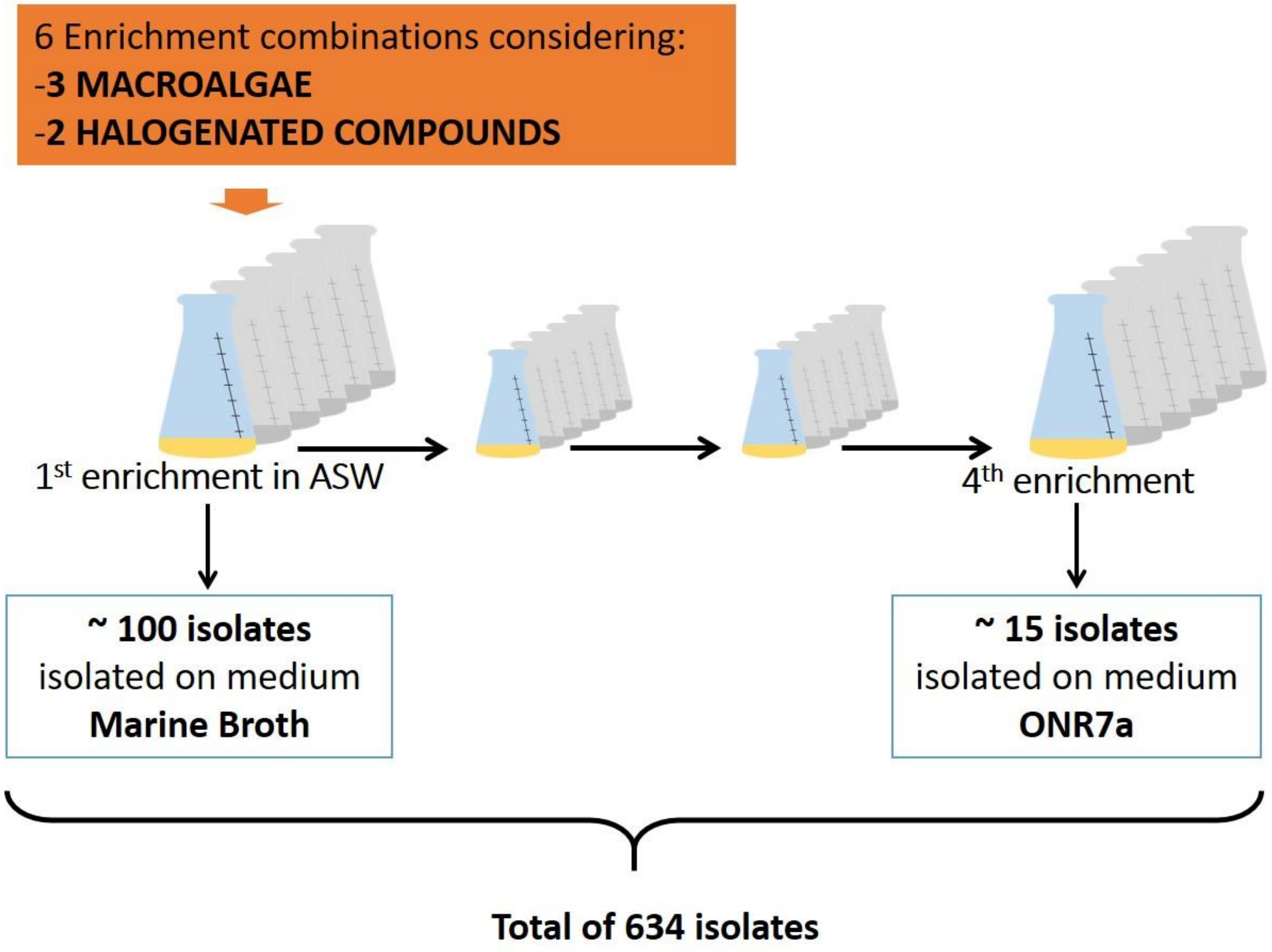

Figure S2 Non-metric MDS distribution of the isolates on the basis of the tested polysaccharidase activities. Isolates are distinguished on the basis of the **A**) alga, **B**) class, and type of activity, *i.e.* **C**) amylase, **D**) pectinase, **E**) alginate lyase and **F**) agarase, considering the class level. In **C-F**) the size of the bubbles indicates the level of the enzymatic activity. Isolates negative for the activity test are not represented.

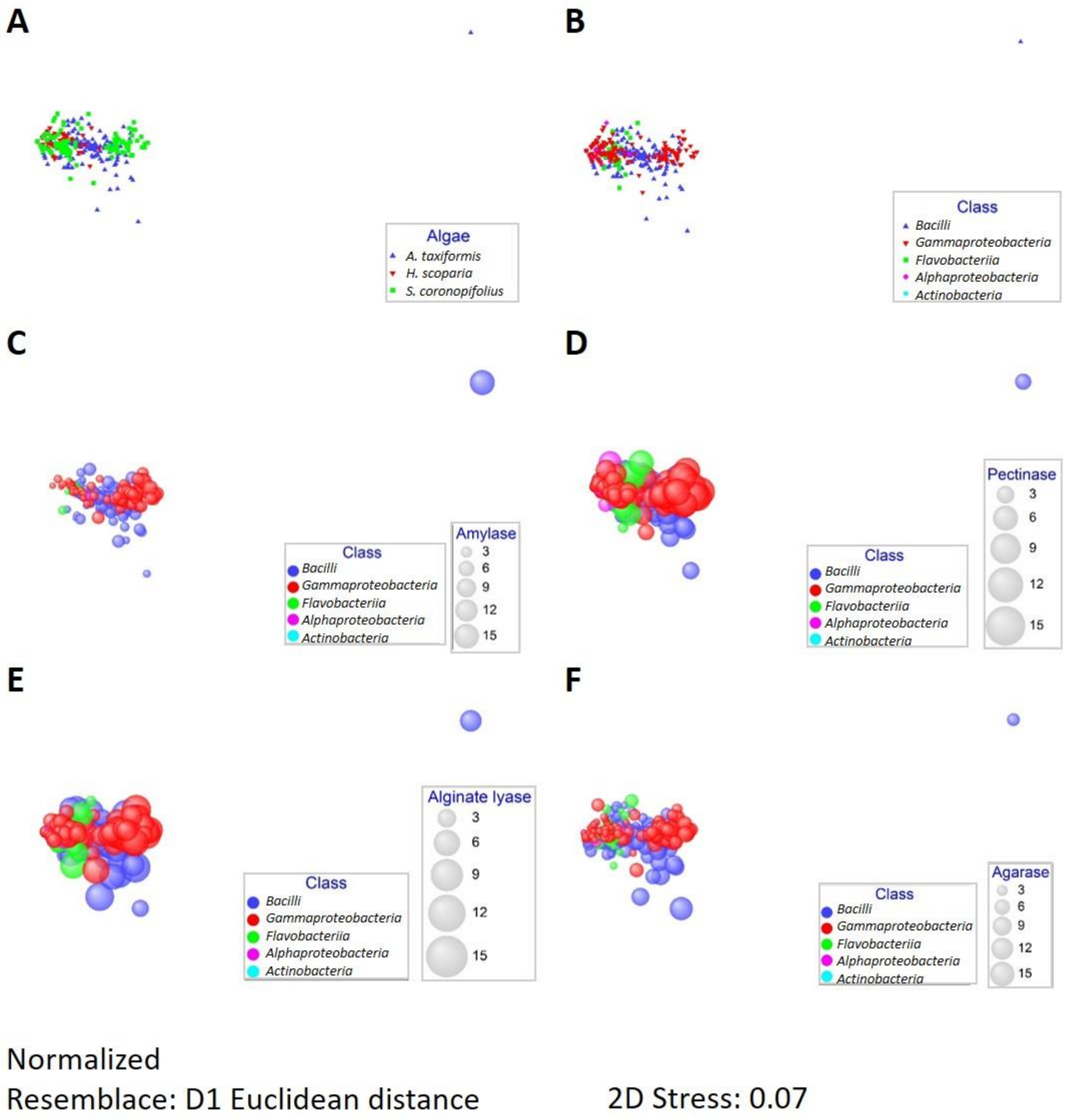

Supplement: Supplementary file 1 — Additional file 1: FigureS1 Schematic representation of the enrichment andisolation process of bacterial strains from decaying algae. The procedure wasapplied to the 3 different macroalgal species with the addition (separately) ofthe 2 different halogenated compounds (1,2-DBE and 1,2-DCA). ASW: artificialseawater. FigureS2 Non-metric MDS distribution of the isolates onthe basis of the tested polysaccharidase activities. Isolates are distinguishedon the basis of the A) alga, B) class, and type of activity, i.e.C) amylase, D) pectinase, E) alginate lyase and F)agarase, considering the class level. In C-F) the size of the bubblesindicates the level of the enzymatic activity. Isolates negative for theactivity test are not represented. [file 13568_2022_1440_MOESM1_ESM.pdf]
